# Editorial: Novel insights into inflammatory roles of mast cells and basophils

**DOI:** 10.3389/fimmu.2023.1186264

**Published:** 2023-03-24

**Authors:** Satoshi Tanaka, Frans J. Van Overveld

**Affiliations:** ^1^ Department of Pharmacology, Division of Pathological Sciences, Kyoto Pharmaceutical University, Kyoto, Japan; ^2^ Department of Science, University College Roosevelt, Middelburg, Netherlands

**Keywords:** mast cell, basophil, inflammation, urticaria, IgE, c-kit, atopic dermatitis

Mast cells and basophils share some phenotypic characteristics, such as the expression of a high-affinity membrane receptor for IgE, FcεRI, and the granule storage of inflammatory mediators including histamine. Mast cells are found to be resident in nearly all vascularized tissues, whereas basophils migrate into the inflamed tissues from the circulation.

Accumulating evidence suggests that cutaneous mast cells play critical roles in chronic spontaneous urticaria (CSU) and that there are several promising therapeutic approaches (1). Sauer et al. verified the involvement of STAT3 in type IIb autoimmune CSU. The roles of histamine in chronic inducible urticaria were reviewed by Kulthanan et al., Takimoto-Ito et al. reported a case in which activated basophils remained in circulation during treatment with omalizumab. Miyake et al. summarized recent findings of human and murine basophils, including their roles in immune tolerance. The regulatory roles of mast cells were also discussed by Zhang et al. and Honda and Honda Keith. Poto et al. and Numata et al. investigated, respectively, the actions of autoantibodies against IgE and sweat antigen-induced chronic activation of basophils in patients with atopic dermatitis. Kamei et al. demonstrated an IgE-dependent murine model of oral allergy syndrome. El Ansari et al. found that allergen-specific IgA could suppress the IgE-mediated activation of mast cells.

MRGPRX2 has received attention as a novel therapeutic target of chronic urticaria and drug-induced anaphylaxis, because its agonists have been found to have a structural diversity (2) (McNeil). In this topic, signal transduction and regulation of intracellular localization of MRGPRX2 were investigated (Chaki et al.; Lazki-Hagenbach et al.). West and Bulfone-Paus discussed the heterogeneity of tissue mast cells, with special attention given to the expression of MRGPRX2. Numata et al. summarized the roles of mast cells in cutaneous diseases. In indolent systemic mastocytosis, an increased number of mast cells expressing MRGPRX2 was found in the skin but was not linked to symptom severity (Pyatilova et al.).

Mast cells have also been found to play important roles in allergic asthma and COPD (3). Rönnberg et al. characterized human lung mast cell populations with a novel approach. Cardenas et al. and Gambardella et al. investigated the effects of alarmins, including IL-33, on mast cells and basophils. Siddhuraj et al. demonstrated the upregulation of CPA3 in lung mast cells upon chronic inflammation. Hellman et al. summarized the *in vivo* functions of mast cell/basophil granule proteases including CPA3. Torres-Atencio et al. reviewed the roles of mast cells in Mycobacterium tuberculosis infection.

Annese et al. and Terhorst-Molawi et al. investigated the roles of mast cells during tumor progression. The proliferation and functions of mast cells are regulated by various factors, including transcription factors, enzymes, and membrane proteins (Sowa et al.; Falduto et al.; Teketomi et al.; Korver et al.; Mishima et al.). Novel murine experimental models for the depletion of mast cells and basophils were also introduced here (Tchen et al.; Hedgespeth et al.).

We hope that this topic encourages and accelerates the research of mast cells and basophils.

## Author contributions

All authors listed have made a substantial, direct, and intellectual contribution to the work and approved it for publication.
